# A Sub-Cellular Viscoelastic Model for Cell Population Mechanics

**DOI:** 10.1371/journal.pone.0012097

**Published:** 2010-08-10

**Authors:** Yousef Jamali, Mohammad Azimi, Mohammad R. K. Mofrad

**Affiliations:** Molecular Cell Biomechanics Laboratory, Department of Bioengineering, University of California, Berkeley, California, United States of America; University of Nottingham, United Kingdom

## Abstract

Understanding the biomechanical properties and the effect of biomechanical force on epithelial cells is key to understanding how epithelial cells form uniquely shaped structures in two or three-dimensional space. Nevertheless, with the limitations and challenges posed by biological experiments at this scale, it becomes advantageous to use mathematical and ‘*in silico*’ (computational) models as an alternate solution. This paper introduces a single-cell-based model representing the cross section of a typical tissue. Each cell in this model is an individual unit containing several sub-cellular elements, such as the elastic plasma membrane, enclosed viscoelastic elements that play the role of cytoskeleton, and the viscoelastic elements of the cell nucleus. The cell membrane is divided into segments where each segment (or point) incorporates the cell's interaction and communication with other cells and its environment. The model is capable of simulating how cells cooperate and contribute to the overall structure and function of a particular tissue; it mimics many aspects of cellular behavior such as cell growth, division, apoptosis and polarization. The model allows for investigation of the biomechanical properties of cells, cell-cell interactions, effect of environment on cellular clusters, and how individual cells work together and contribute to the structure and function of a particular tissue. To evaluate the current approach in modeling different topologies of growing tissues in distinct biochemical conditions of the surrounding media, we model several key cellular phenomena, namely monolayer cell culture, effects of adhesion intensity, growth of epithelial cell through interaction with extra-cellular matrix (ECM), effects of a gap in the ECM, tensegrity and tissue morphogenesis and formation of hollow epithelial acini. The proposed computational model enables one to isolate the effects of biomechanical properties of individual cells and the communication between cells and their microenvironment while simultaneously allowing for the formation of clusters or sheets of cells that act together as one complex tissue.

## Introduction

One of the important phenomena in cell engineering and developmental biology is the shape of tissue and the cell's organization. Depending on the cell type and environmental conditions, cells can create unique shapes such as flat sheets, self-enclosed monolayers, cysts, or elongated tubes. The more important question is how these cells interact and how their local interaction causes a global geometrical distinctive shape for tissues like the heart or kidney [1]–[6]. The geometrical interactions and coordinated adhesion among neighboring cells and between the cells and local environment are critical for structure and function of epithelial tissue [7]–[13]. Any perturbation of these orchestrated interactions can cause abnormality in behavior and function of tissue and often lead to initiation of tumor growth and invasion [14]–[15]. Another interesting subject is embryogenesis, when a stem cell with consecutive rapid divisions and differentiation can create different tissues, wherein the interactions between cells and environmental biochemical and biomechanical signals have critical, yet nearly unknown roles [16]–[17]. However, in the last two decades, improved experimental techniques and developments in new laboratory instruments have allowed for more detailed understanding of cell-cell communication and the cell's response to biochemical and biomechanical environmental signals. Nevertheless, biological experiments are expensive and depend on many parameters that are mostly difficult to control and test in isolation. As a complementary method, mathematical modeling and ‘*in silico*’ (computational) experiments are a good candidate to help explore the behavior of the individual tissue cells along with investigating their response to environmental cues. Due to easy isolation in *in-silico*, computational models, incorporating the related fundamental physical and biological parameters can explain how specific biochemical or biomechanical parameters may affect the tissue cells and their arrangement. Such a model can help reduce the number of experiments required to obtain meaningful observations by eliminating unlikely hypothesis while providing a better explanation of observations.

**Figure 1:**
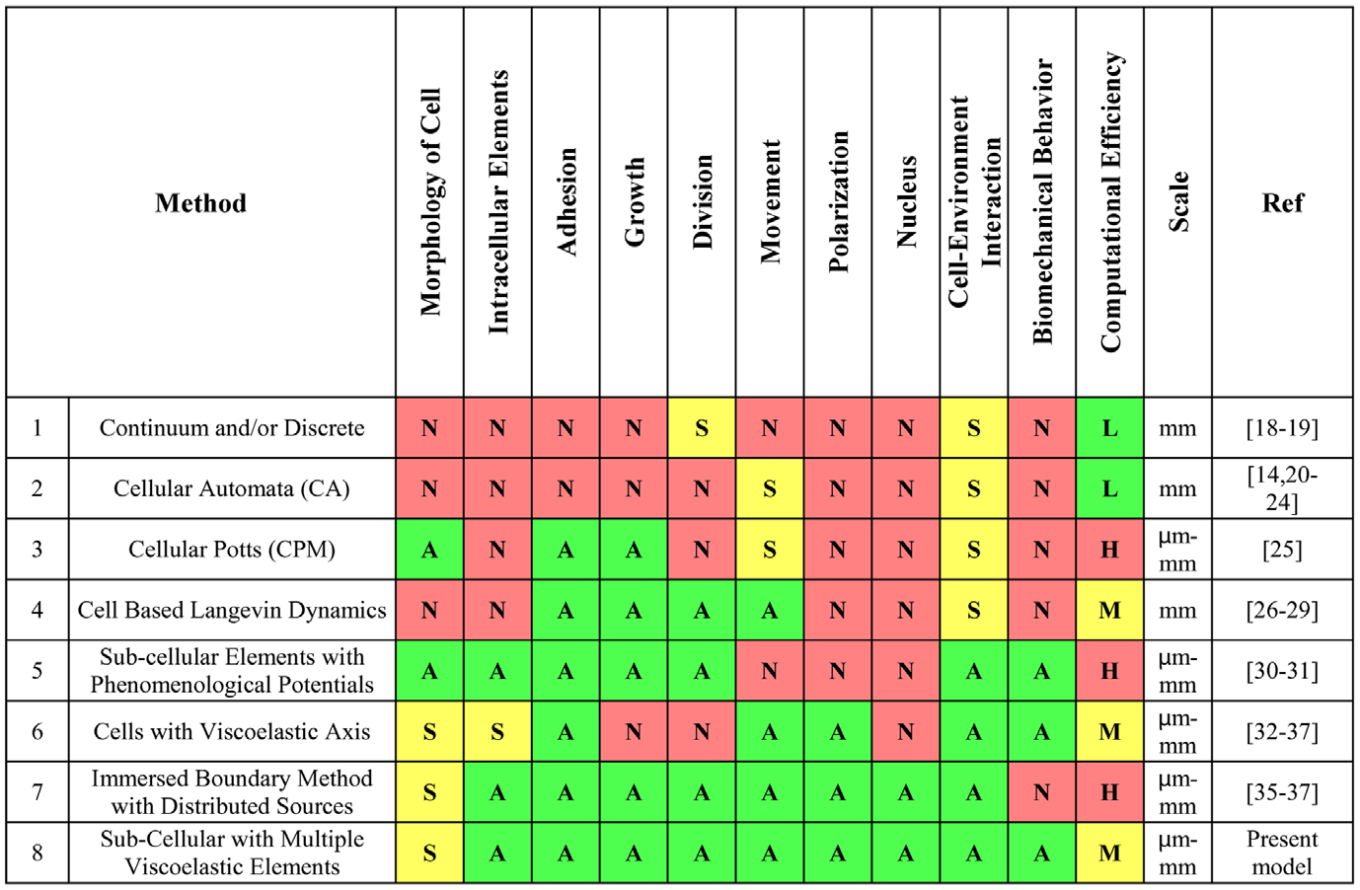
A comparison of existing models for cell morphology based on model realism and computational cost. A = Advanced, S = Simple, N = None; L = Low, M = Moderate, H = High.

For example, to investigate how individual cells cooperate and contribute to the overall structure and function of a particular tissue, a proper computational model must be capable of allowing cells to be defined as individually deformable shapes, time- and space-dependent individually regulated cell turnover, and cell-cell and cell-ECM interaction. Many models have been developed to mimic cell behavior, such as response to external mechanical and biochemical signals, cell-cell interaction, cell motility, and cell morphology. For example, some models have attempted to mimic cell collection behavior such as cancer invasion through the use of continuum and/or discrete approaches [18]–[19] (Figure 1; Method 1), where each cell is represented by a finite element and follows a cellular automata (CA) method [14], [20]–[24]. Several models are based on the extended Cellular Automata method (Figure 1; Method 2), e.g. the lattice-gas based cellular automata (LGCA), and the cellular potts model (CPM) [25] (Figure 1; Method 3). In other approaches, cells are modeled as colloidal objects capable of interacting with their environment [26]–[29]. In such models, cells are capable of migrating, growing, dividing, and changing their orientation. For example, in a model proposed by Galle, Loeffler et al. (2005), the cells move according to the Langevin dynamics framework and can interact based on a combination of attractive and repulsive forces (Figure 1; Method 4). The aim of these models is to replicate the multi-cellular growth phenomena. By focusing on monolayer culture, Galle et al. [29] have investigated the effect of key factors on rate and quality of culture growth. They also analyzed the underlying processes involved in multi-cellular spheroids, intestinal crypts, and other aspects of developmental biology. These models are robust in mimicking various aspects of cell population, but fail to examine the effects of cell deformation and morphology on pattern formation and growth processes. To investigate the effects of cell morphology on a multi-cellular structure, Newman and colleagues [30]–[31] developed a phenomenological model involving a number of identical sub-cellular elements, whose dynamics and interactions are defined by intracellular potentials, which are stronger and bind elements belonging to the same cell as well as intercellular potentials, which are weaker and bind elements of neighboring cells (Figure 1; Method 5). This model can simulate cell growth and division, and when modeling the growth of a multi-cellular cluster from a single cell, this algorithm simulates cellular shapes and multi-cellular structures in 3D. Some models are based on the viscoelasticity of cells [32]–[37], where each cell includes certain elastic and viscous elements. Such models lend themselves to easy incorporation of the cell-cell adhesion and repulsion, and various forces acting on individual cells in the cluster. For example, a 3D deformable cell model with cell adhesion and signaling was developed by Palsson and colleagues [32]–[33], where each cell is taken as an ellipsoid, with its axis composed of a combination of springs and viscous elements (Figure 1; Method 6). This model was used to investigate the role of cell signaling, cell adhesion, chemotaxis, and coordinated differentiation in the morphology of a developed organism. Another biomechanical approach developed by Rejniak and colleagues [35]–[37] represents cells as deformable viscoelastic objects that can be arranged into tissues of various topologies. Rejniak's model employs an immersed boundary method with distributed sources (Figure 1; Method 7). This approach joins elastic cell dynamics with a continuous representation of a viscous incompressible cytoplasm. The model covers many aspects of cellular behavior such as cell growth, division, apoptosis and polarization. With this model it is possible to investigate the biomechanical properties of cells and cell-cell interaction, the effects of the microenvironment on a cellular cluster, and how individual cells work together and contribute to the structure and function of a particular tissue. Some applications of this model include the following: the folding of a trophoblast bilayer [38], tumor growth [35] and self-arrangement into a hollow acinus [36]. More recently, Coskuna et al. [34] developed a mathematical model for ameboid cell movement in which a viscoelastic (spring–dashpot) system was used to represent the cytoskeleton. This model was used to solve an inverse problem of amoeboid cell motility and to find the variation of spring and dash-pots parameters in time. The research shows that the model and the solution to the inverse problem for simulated data sets are highly accurate. In general, cell mechanics has been modeled based on non-living structures using different approaches [39] ranging from the soft glassy material model [40]–[41], to the cortical shell–liquid core model [42]–[43], and tensegrity architecture [44]–[51]. Few of the theoretical models that have been proposed for analyzing the mechanical properties of adherent living cells are capable of simultaneously incorporating (i) the discrete nature of the cytoskeleton, (ii) cell–cell and/or cell–extracellular matrix (ECM) interactions, and (iii) the cellular pre-stress [48]. To address this shortcoming, a new biomechanical model for the cell is proposed here that is able to incorporate all these properties of the cell simultaneously. In this model, inspired by the tensegrity concept, each cell is capable of changing its morphology, and performing various cellular processes such as growth, division, death, and polarization. The modeled cells are able to interact with each other and with their environment. Each cell in this model is an individual unit containing several subcellular elements, such as the elastic plasma membrane, encompassed by viscoelastic elements that perform the function of the cytoskeleton, and the viscoelastic elements of the cell nucleus (Figure 1; Method 8). Additionally, the cell membrane is divided into segments where each segment (or point) incorporates the cell's interaction and communication with its environment, such as adherens junctions. The qualities of the various models discussed above are summarized in Figure 1. The remainder of this paper is organized as follows. In Methods, the mathematics of the model have been discussed. In Result and Discussion, the model has been used to mimic several relevant biological examples to show validity and the capabilities of the model. In Conclusions, the model and its capabilities are summarized and future steps for improving the model have been suggested.

## Methods

**Figure 2:**
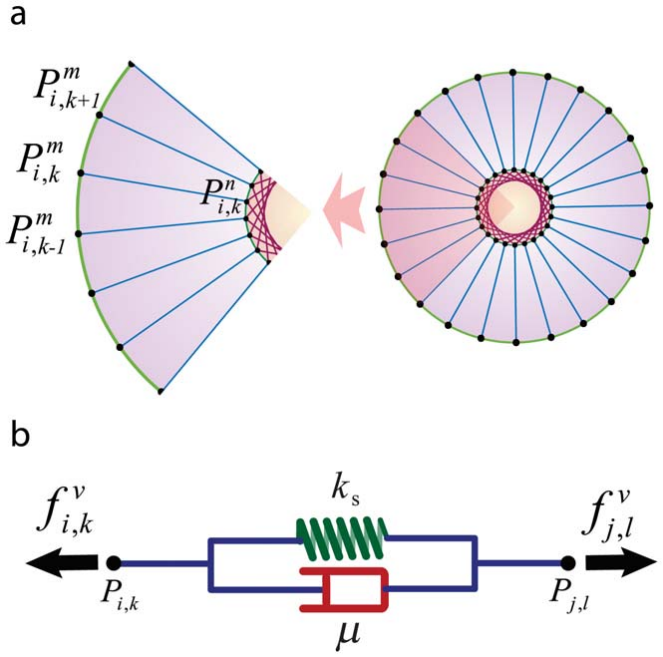
Cell structural model. a) The perimeter of the cell and nucleus (i.e. their corresponding membranes) are initially discretized into 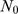 nodes (points). The superscript 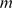 indicates that the point is on the cell membrane and superscript 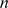 represents a point on the nuclear membrane. If neither 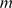 nor 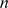 are specified, the given point can be assumed to lie on either the cell membrane or nucleus. For example, the 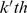 membrane's point of 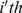 cell represented by 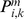. Each line that connects two points (red, green and blue lines) refers to a Voigt subunit. The total force that acts on each point is 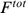 and is calculated by Eq(1) b) Voigt subunit. A linear Kelvin-Voigt solid element, represented by a purely viscous element (a damper) and purely elastic element (a spring) connected in parallel. The force that is exerted on 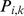 from this subunit is 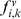 (Eq.(2)). 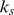 is the spring constant and 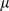 represents viscosity.

The details of our computational model are outlined here while Table 1 summarizes the parameters of this paper and Table 2 provides quantitative values that were used in this model that are valid for a typical cell. In this model, cells are represented as objects with initial circular structures. The cell and nuclear membranes are initially discretized, arbitrarily, into 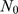 nodes (points), where a mass is associated with each point representing altogether the total mass of the nucleus and cytoskeleton. Each point on the cell and nuclear membranes is then 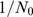 of the mass of the cytoskeleton and nucleus, respectively (Figure 2). Hereafter, unless explicitly mentioned, the subscripts of parameters refer to the cell number and point number. Superscript 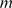 indicates that the point is on the cell membrane, and superscript 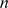 represents a point on the nuclear membrane. If neither 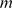 nor 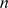 is specified, the given point can be assumed to lie on either the cell membrane or nucleus. For example, the 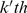 membrane point of the 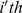 cell is represented by 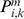, the corresponding point in the nucleus is represented by 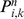, and 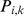 represent the general point 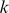 of 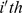 cell.

**Table 1:** List of variables and their definitions used in this paper.

Subscripts		Parameters	
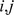	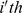 and 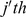 cells	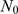	number of nodes (points) on the perimeter of the cell and nucleus (i.e. their membranes)
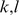	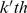 and 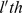 points	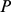	point
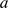	anterior region of cell	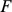	force
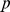	posterior region of cell	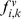	force of a Voigt subunit acting on 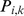
		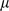	viscosity of a Voigt subunit
		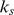	spring constant of a Voigt subunit
		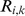	position of 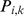
Superscripts		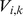	velocity of 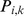
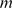	cell membrane	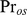	inner pressure
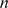	cell nucleus	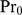	environmental pressure
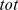	total of all related parameters	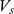	stop volume region
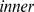	inner cell	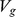	growth volume region
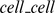	cell-cell interactions	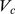	current volume of the cell
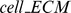	cell-ECM interactions	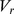	rest volume of the cell
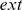	external parameters	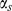	stop volume region coefficient
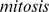	mitosis process	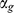	growth volume region coefficient
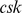	cytoskeleton	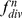	dividing force that acts on nucleus points
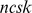	nucleus cytoskeleton	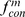	dividing force that acts on membrane points
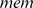	membrane	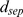	distance separating two daughter cells
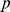	pressure	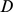	drag coefficient of each point
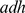	adhesion	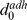	maximum separation between two points for initiating adhesion
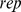	elastic cell-cell or cell-ECM repulsion	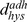	minimum separation between two points for disrupting adhesion
		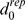	maximum separation between two points for elastic interaction
		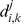	distance between 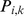 and the membrane surface of 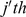 cell or ECM
		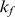	form factor
		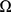	total occupied area of cells
		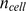	number of cells
		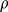	cell edge density
		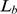	sum of the lengths of all internal cell boundaries plus half of the perimeter of the patch

**Table 2:** The parameters for a typical cell, most of the parameters adopted from [34].

P	Definition of Parameters	Units	Value
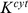	Radial spring constants	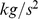	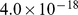
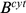	Damping constants for radial springs	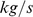	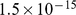
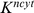	Nucleus spring constants	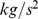	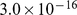
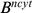	Damping constants for nucleus springs	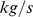	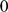
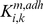	Adhesion spring constants	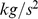	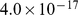
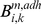	Damping constants for adhesion spring	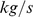	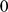
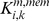	Cell membrane spring constants	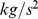	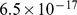
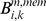	Damping constants for cell membrane springs	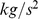	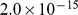
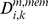	Drag coefficients at the cell membrane	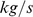	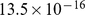
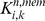	nucleus membrane spring constants	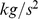	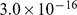
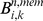	Damping constants for the springs	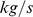	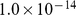
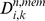	Drag coefficients at the nucleus membrane	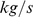	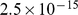
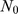	Number of initial points		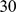
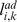	Adhesion spring rest lengths	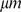	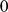
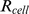	Cell radius	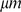	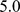
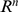	Nucleus radius	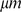	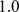
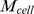	Cell mass	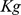	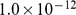
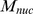	Nucleus mass	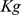	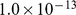
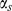	Percent of rest volume where growth ends under this volume		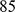
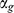	Percent of rest volume that growth continues above this volume		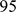
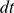	Time step	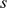	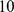
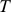	Temperature	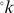	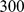

**Figure 3:**
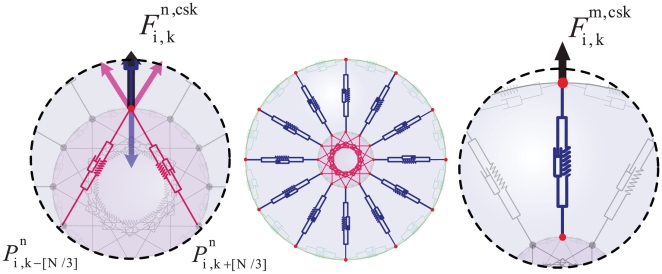
Inner cell structure and forces. The mechanical properties of the cytoskeleton are modeled using Voigt subunits; the spring constants of the model are linear approximations to the elasticity of the inner cell. All springs can be considered subject to a damping force due to the viscosity of the cytoplasm, where linear dash-pots are used to approximate the viscosity of the cytoskeleton. In our model, the cytoskeleton is divided into 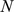 uniformly radial distributed parts, each of which is replaced by a Voigt subunit radiating from the nucleus (blue subunits). Each subunit connects two points of the cell and nuclear membrane, which are located at a radial direction from the center of the nucleus. The model also contains 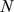 Voigt subunits in the nucleus (red subunits), each of which connect two nuclear membrane points 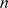 and 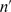 in which 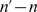 equal to 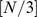, This allows the nucleus to show more resistance to changes in its shape and volume due to exterior pressure. 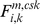 is the cytoskeletal force acting on 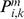 and is calculated by Eq. (3). 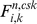 is the force acting on 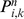 from the cytoskeleton and nuclear cytoskeleton and is calculated by Eq. (4).

**Figure 4:**
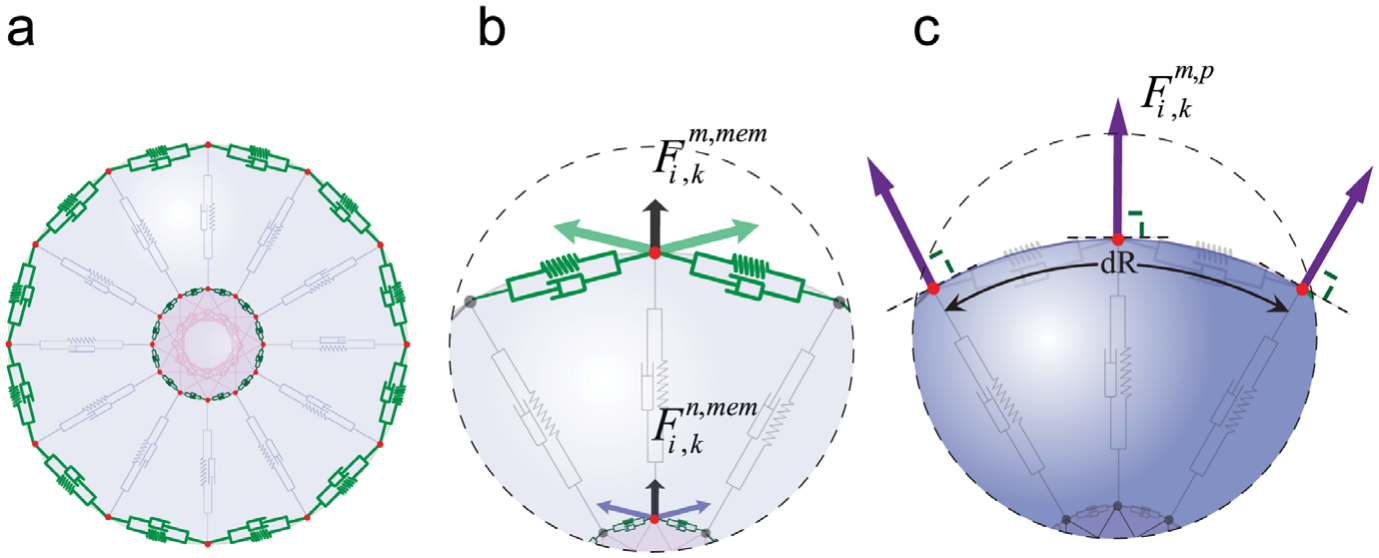
Structure of cell membrane and cytoplasm. a,b) To represent the viscoelasticty of the membrane and cortical cytoskeleton, two consecutive membrane points are connected with a Voigt subunit (green subunits); hence, the model includes 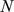 Voigt subunits on the cell membrane and 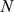 subunits on the nuclear membrane. The forces acting on each cell from membrane subunits is calculated by Eq. (5); as the figures show, each point is subject to two adjacent subunits. c) An osmotic pressure will act on the membrane. This internal pressure is involved in cell morphology and affects the driving force of cell movement [57]–[60]. Knowing the persistence lengths of micotubles, and the fact that they appear curved in the cell, it follows therefore, that this filament pushes the membrane outward [39]. Therefore, a pressure field acting upon each point of the cell membrane, representing cytoplasmic pressure with an outward and perpendicular direction to the cell membrane can be defined as 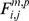 by Eq. (6).

**Figure 5:**
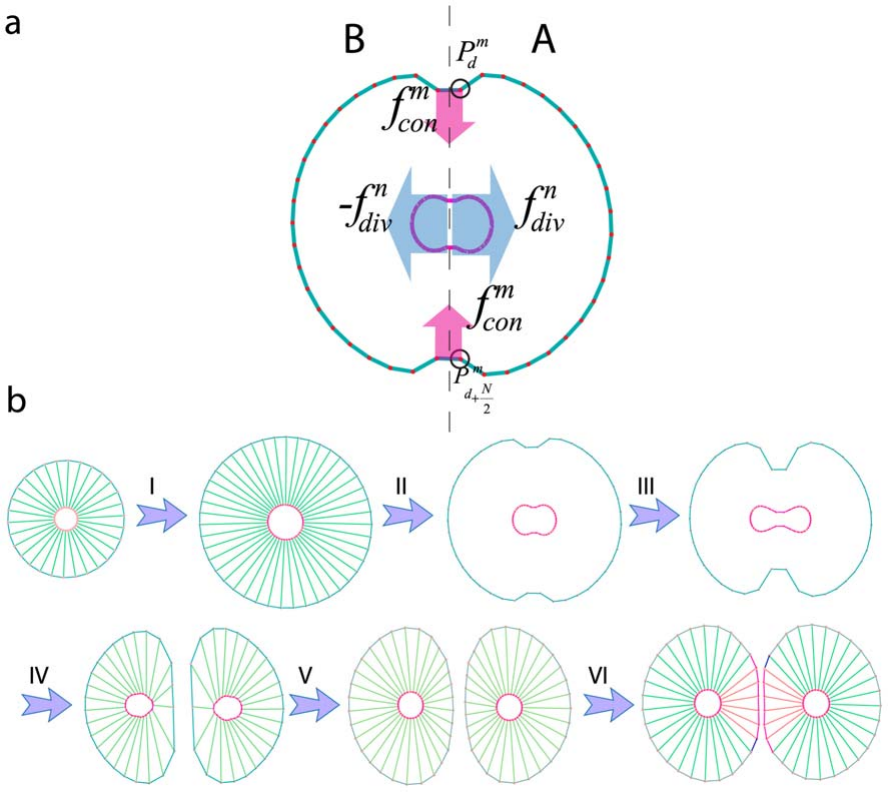
Mitosis and involved forces. a) Several bio-mechanical aspects of cell proliferation are included in our model. First, the cell area (or volume in 3D) is doubled. The axis of cell division is selected (dash-line), in a dividing unpolarized cell, this axis usually is perpendicular to the cell elongation direction in such a way as to split the cell into two approximately equal parts. In a partially polarized cell, however, the axis of cell division is orthogonal to the part of the cell membrane that is in contact with the ECM. Two new daughter nuclei are then placed orthogonal to the axis of cell division. After the selection of division axis, the model finds the nearest membrane point to this axis, i.e. point 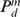 In mitosis there are two major mechanical forces, first in the Anaphase stage of mitosis, shortening the spindle fibers caused by the kinetochores separation, and the chromatids (daughter chromosomes) are pulled apart and begin moving to the cell poles [65]. Second, a contractile ring is formed by contractile forces acting on the opposite sites of the cell boundary in the cytokinesis process [65]. This results in the formation of a contractile furrow and causes division of the cell into two daughter cells. The cell points can therefore be divided into two groups, A and B, where group A consists of membrane points from 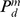 to 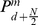 and nucleus points from 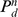 to 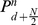 and the remaining points belong to group B. To model the first mechanical force, the points of the nucleus in A and B are pulled apart, in the orthogonal direction to the division axis with force 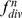 (Eq.(8) ). During the nucleus separation, the contractile force, 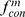, acts on boundary points of A and B groups to model the second mechanical force(Eq. (9)). b) The main phases of cell growth and division. (I) Cell growth. To implement cell growth in the proposed model, the number of membrane points, i.e. the number of viscoelastic compartments, is allowed to increase. When we add two points on each the cell and nuclear membranes, four subunits are added to the system, with the parameters of these new subunits calculated from the average of the first neighbor's homogeneous subunit parameters. With the additional ‘growth’ point, the circumferential length of the membrane increases in proportion to 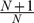. Hence, the rest volume i. e. the volume of the cell when it grows freely without any inner or outer constraint, must increase proportional to 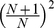. Therefore, the rest length of radial springs is increased in proportion to 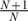. When the area (or volume) of cell doubles, the number of defining membrane points increases to 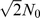, where 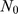 is the number of membrane points on the initial cell. (II,III) Mitotic process: two types of forces act on points to divide the cell. Due to these forces the cell elongates and prepares for division. (IV) Two new daughter nuclei are then placed orthogonal to the axis of cell division. After the nucleus separates, i.e. the distance between the center of the mass of nuclear points exceeds a certain value, 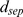, the cell will divide into two daughter cells, i.e. the subunits which join the boundary points will be eliminated and will bind to a new first neighbor point in the same group with a new subunit. V) After division takes place, each daughter cell will only have 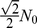 points, and as a result it is possible to simultaneously add 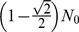 points to each cell. To add membrane points, two consecutive points in the membrane are found that have the longest distance and a new point is inserted between them, and this process is repeated until the number of cell points becomes 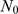. f) Adhesion of the two daughter cells.

Force balances at each point have been considered as follows. The total force 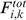, acting on 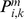 is calculated according to
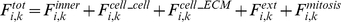
(1)Here 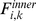 is the total force that acts on 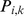 due to the inner structure of the cell (Figure 3 and Figure 4), i.e. the cytoskeleton, membrane, and the cytoplasm, 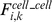 is the total force resulting from the interaction with other cells (cell-cell interaction), 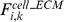 is the force due to an interaction with a substrate(cell-ECM interaction), 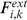 represents the external forces, (as described below) and 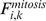 is the sum of the forces acting on 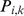, when the cell undergoes the cell division process (Figure 5). This force is due to the shortening of the spindle fibers; a contractile ring is formed by contractile forces acting on opposite sides of the cell boundary. In the following subsections, these forces will be discussed and described further in the context of various cellular events.

### a. Inner cell structure

To model the inner cell structure, a viscoelastic Voigt model, represented by a purely viscous element (a damper) and purely elastic element (a spring) connected in parallel, is used (see Figure 2). The force of a Voigt subunit connected to points 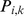 and 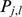 is given as:
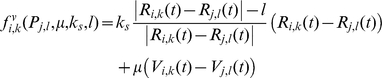
(2)Here 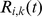 and 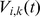 are the position and velocity corresponding to 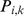 respectively, 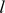 is the rest length of the spring, 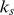 is the spring constant, and 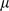 represents viscosity. It should be noted that 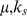 and 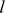 vary with time and position and depend on *i^th^*, *j^th^* cells and *k^th^*, *l^th^* points, i.e. the two points that are connected by this element (see Figure 2). The inner force 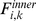 (see Figure 3 and Figure 4) can be represented by the equation:
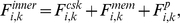
where 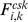 is the force from the cytoskeleton, 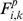 is the force due to liquid in the inner cell, i.e. cytoplasm, and 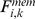 is the force from the membrane acting on 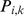.

#### Cytoskeleton

The mechanical properties of the cytoskeleton, like elasticity and viscosity, are critical to the validity of the model. Voigt subunits are effective for modeling a viscoelastic system; the spring constants of the model are linear approximations to the elasticity of the inner cell. Additionally, all springs are subjected to a damping force resulting from the viscosity of the cytoplasm, where linear dash-pots are used to approximate the viscosity of the cytoskeleton. In the present model, the cytoskeleton is divided into 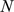 uniformly radial distributed parts, each of which is represented by a Voigt subunit radiating from the nucleus (Figure 3, blue subunits). Each subunit connects two points of the cell and nuclear membrane, which are aligned in a radial direction from the center of the nucleus. The nucleoskeleton is represented as a viscoelastic model involving an actomyosin system (Figure 3, red subunits). The model also contains 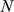 Voigt subunits in the nucleus (Figure 3, red subunits), each of which connects two nuclear membrane points 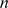 and 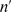 in which 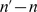 equal to 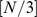 (Figure 3). This allows the nucleus to show more resistance to changes in its shape and volume due to exterior pressures when compared to simply connecting opposing points on the cell membrane. Only elements from the cytoskeleton act on each point in the cell membrane, therefore, it can be said (see Figure 3):
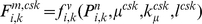
(3)For nuclear membrane points (see Figure 3):
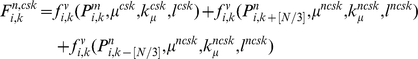
(4)Where 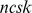 refers to the nuclear cytoskeleton.

As a note, the inclusion of additional cellular elements into a biomechanical model should be justified. In our model, an additional yet key biomechanical element that is considered is the structure of the nucleus' cytoskeleton (actin filaments [52] and nuclear lamina) which is connected to the cytoplasm cytoskeleton via connected proteins (LINC) [53]. Our model attempts to incorporate key aspects of the cell that play an important role in cell biomechanics while maintaining simplicity. Additionally, not all cells are flat nor are their nuclei positioned in the center of the cell. The fact that the nucleus is not positioned in the center of the cell plays an important role in the shape of the cell (e.g. satellite shape of fibroblasts). Furthermore, under some mechanical conditions, the nucleus plays an important role in the final shape of the cell. Moreover, the mechanical behaviors of the nuclear region (e.g. kinetochore microtubule shortening) play a key role in mitosis. Inclusion of the nucleus in the model helps substantially in modeling the dynamics of mitosis based on what happens in the real cell during this process. In cellular mechanotransduction, the nucleus itself may play an important role in the response of the cell to force [54] and the forces acting on the nucleus are believed to be important in eliciting events such as gene expressionas shown by Wang *et al* [55]. Subsequently, through inclusion of the nucleus in our model, we can investigate the effect and intensity of forces that act on the nucleus from the external environment through the cytoskeleton. The inclusion of the nucleus is ultimately necessary in multi-scale modeling of the cell. From a modeling point of view, if we were to ignore the nucleus, we would need to connect all of the end points of the cytoskeleton elements to each other at a single central point. In this situation this point will play a critical role in simulations and can cause some singularities and abnormal behavior during simulation and impose many limitations on the model. Conversely, with the current structure, the force is distributed around the nucleus and the whole system is more stable (A possible alternative is to connect each point on the membrane to the point on the opposing side of the membrane; in this case each force on one point is directly transmitted to the other side of the cell and causes artificial behavior).

#### Membrane

To represent the viscoelasticty of the membrane, or, more correctly, the viscoelasticty of the cortical cytoskeleton, two consecutive membrane points are connected with a Voigt subunit (Figure 4, a and b, green subunits); hence the model includes 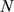 Voigt subunits on the cell membrane and 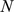 subunits on the nuclear membrane.
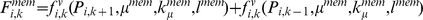
(5)


#### Cytoplasm

The cytoplasm is a viscous incompressible fluid, naturally hindering the cell's shape and volume changes. On the other hand, osmotic pressures caused by the relatively higher concentrations of proteins and other molecules inside the cell compared to its external environment [56] act on the membrane. This internal pressure is involved in the determination of cell shape and morphology and affects the driving force of cell movement [57]–[60]. In addition, the curved shape of microtubules in the cell (despite their large effective persistence length compare to the length of the cell [39], [61]) implies that they must push the cell membrane outward [39]. To represent these, a normal stress (pressure) field acting on each point of the cell membrane is defined, whose direction is outward and perpendicular to the cell membrane (Figure 4c):
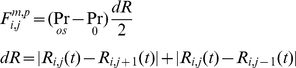
(6)where 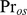 and 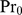 are the inner pressure and environmental pressure, respectively. However, we make the approximation that pressure in the cell is constant and independent of volume in equilibrium conditions. This approximation is not very speculative as various membrane channels allow for the flow of intracellular fluid into or out of the cell at equilibrium, not allowing for the buildup of hydrostatic pressure due to intracellular fluid accumulation.

### b. Cellular processes

#### Growth

To implement cell growth in the proposed model, the number of membrane points, i.e. the number of viscoelastic compartments, is allowed to increase as follows. First an integer random number in the range 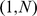 is generated, say 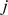, providing the location of the point 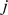 on the perimeter, then a point between 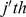 and 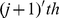 points in the cell membrane is added and the same is done for the nuclear membrane. As a result, four subunits are added to the system, one Voigt subunit for each the cell membrane, nuclear membrane, inner nucleus, and cytoplasm). The parameters of these new subunits are calculated from the average of the first neighboring homogeneous subunit parameters. With the additional ‘growth’ point, the circumferential length of the membrane increases in proportion to 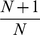. Hence, the rest volume (in 2D), i. e. the volume of cell when it grows freely without any inner or outer constraint, must increase proportional to 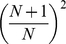, therefore the rest length of radial springs is increased proportionally to 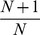. Two regions of volume, stop volume region, 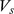, and growth volume, 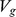 are defined as:
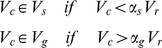
(7)Where 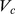 is the current volume of the cell, 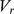 is the rest volume (as defined above), 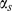 and 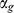 are the coefficients that define the extrema of these regions (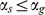). Often, due to external pressures and environmental space limitations, notwithstanding cell growth, the cell's volume cannot increase and will hence fall in the stop volume region, i.e. the cell ceases to grow. Under this condition, the cell cannot continue its growth until its volume fills the available space.

#### Mitosis

The present model allows for a growing cell to divide, provided that its volume falls in the growth volume region (see definition above). Key biomechanical aspects of cell proliferation are included in our model. When the cell area (or volume in 3D) is doubled, the axis of cell division is selected, so that the orientation depends on the cell shape, extracellular environment and cell polarization status [62]–[64]. In a dividing unpolarized cell, this axis, usually perpendicular to the direction in which the cell elongates, causes the cell to split into approximately two equal parts. In a partially polarized cell, however, the axis of cell division is orthogonal to the part of the cell membrane that is in contact with the ECM. Two new daughter nuclei are then placed orthogonal to the axis of cell division, (see Figure 5). This axis must include the center of mass of the nucleus. After the selection of division axis, the model finds the nearest membrane point to this axis, i.e. point 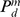 (Figure 5a). As the structure of the nucleus, during mitosis collapses [65], the nuclear subunits in our model will be eliminated during mitosis, followed by the formation of new nuclear subunits for the daughter cells. In mitosis there are two major mechanical forces that occur. First, during the anaphase stage of mitosis, the shortening of the spindle fibers causes the kinetochores to separate and the chromatids (daughter chromosomes) to be pulled apart and to begin moving toward the poles of the cell [65]. Secondly, during the cytokinesis process, a contractile ring is formed by contractile forces acting upon opposite sides of the cell boundary [65]. This results in the formation of a contractile furrow and causes division of the cell into two daughter cells. In our model, the cell points are divided into two groups, A and B, where the A group consists of membrane points from 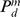 to 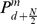 and nucleus points from 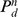 to 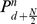 and the remaining points belong to group B, see Figure 5a). To model the first mechanical force, the points of the nucleus in A and B are pulled apart, in an orthogonal direction of axis division with the force 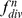.
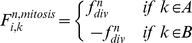
(8)During the nucleus separation, the contractile force, 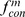, acts on the boundary points of groups A and B to model the second mechanical force. That is,
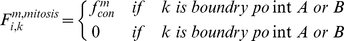
(9)After the nucleus is divided, i.e. the distance between the center of mass of the nucleus points in groups A and B exceed a certain value, 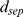, the cell will be divided into two daughter cells i.e. the subunits which join the boundary points of A and B are eliminated and bind to new first neighbor points in the same group with a new subunit (see Figure 5).

When the area (or volume) of the cell doubles, the number of defining membrane points increases to 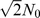, where 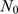 is the number of membrane points on the initial cell. After division takes place, each daughter cell will only have 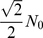 points, and as a result it is possible to simultaneously add 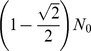 points to each cell. To add membrane points, two consecutive points in the membrane are found that have the longest distance and insert a new point between them, and repeat this process until the number of cell points become 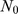.

The main phases of cell growth and division are presented in Figure 5b.

#### Motility

**Figure 6:**
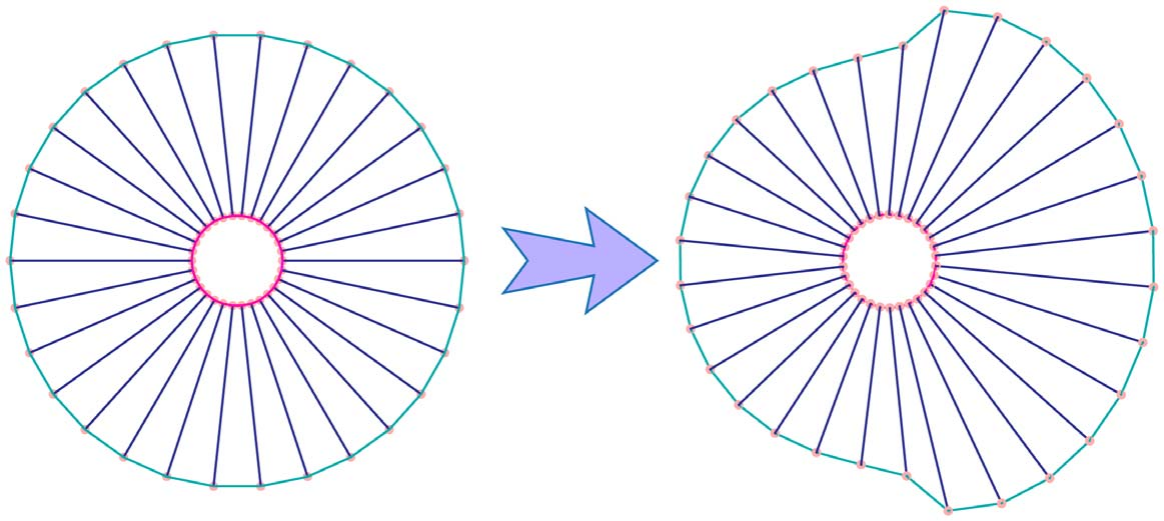
Motility. Cell crawling is generated by the interplay between three different processes, namely, protrusion, adhesion, and contraction. These processes cooperate in a spatially heterogeneous structure to generate a complex topology for cell motion while correlation and coordination between them has a significant role on the motility of the cell [66]. To model these three-stage events, the cell is first polarized by categorizing the points into two groups, the anterior and posterior, where their cytoskeleton subunit parameters will change periodically in a coordinated fashion. In addition to modeling the adhesion with a substrate, the drag coefficient is used for each of the points which will change periodically in coordination with variation of subunit parameters. The method for the variation of the parameters is shown in Eq.(10).

Cell motility is an important biological phenomenon that plays a key role in morphogenesis, metastasis, and wound healing [66]. Cell motility involves the interplay between three different processes, namely, protrusion, adhesion, and contraction. Protrusion occurs during the process of cytoskeletal assembly where the cell front pushes out the cell's leading edge. Next, adhesion occurs with the extracellular environment, whereby the cell establishes adhesion to the surface at the front end and slowly retracts from the back end. Finally, contraction of the actomyosin filaments causes the rest of the back end of the cell to pull up. These processes cooperate in a spatially heterogeneous structure to generate a complex topology for cell motion and correlation. Coordination between these processes has a significant role on the motility of the cell [66]. To model these three-stages, the cell is first polarized by categorizing the points into two groups, anterior and posterior. The cytoskeletal subunit parameters of these stages will change periodically in a coordinated fashion. In addition, to model the adhesion with a substrate, the drag coefficient for each point is used. These points will also change periodically in coordination with various subunit parameters, as follows:
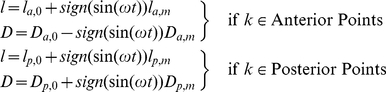
(10)

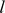
 is the rest length of cytoskeleton subunits spring, the subscript 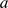 and 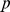 refer to the anterior and posterior region, subscript 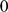 shows the initial value and subscript 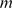 refers to the threshold of parameters in motility, for example 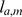 shows the threshold of the spring rest length that is anterior of the crawling cell. 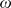 is the variation frequency of parameters in the posterior and anterior position. 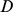 is the drag coefficient of each point (Figure 6) and its value is proportional to the number of adhesions. This idea of relating the number of adhesions to the drag coefficient has been used in other models of cell motility [67]–[69]. The variation of 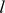 represents the protrusion and retraction in the cell that is formed by actamyosin systems.

#### Apoptosis

**Figure 7:**
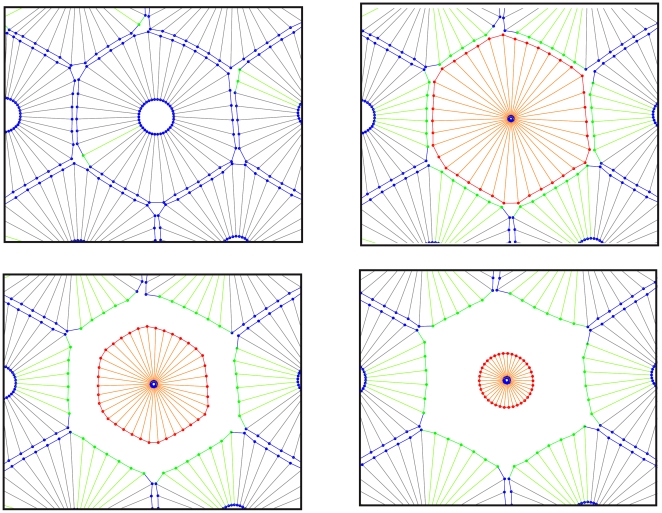
Apoptosis. The structure and morphology of apoptotic cells undergo dramatic changes, including detachment from the neighboring cells, collapse of the cytoskeleton, shrinkage of the cell volume and alternations in the cell surface. Apoptosis progresses quickly and its products are quickly removed. To model these events, cell adherens junctions with their neighboring cells and/or substrate are initially disassembled (top right). Then, the spring constant length of all subunits are reduced arbitrarily and the inner pressure is removed (bottom left), so the cell will collapse and the cell area is gradually reduced until it reaches a prescribed minimal value (bottom right). At this time, the cell is considered to be dead and will be removed from the system.

The structure and morphology of apoptotic cells show the cell undergoing dramatic changes, including detachment from the neighboring cells, collapse of the cytoskeleton, shrinkage of cell volume and alterations in the cell surface resulting in an irregular bulge in the plasma membrane, called bleb [65]. The process of apoptosis progresses quickly and its products are removed immediately. To model these events, cell adherens junctions first disassemble with the neighboring cells and/or substrates. Then, the rest length of each subunit spring is reduced arbitrarily (five-fold in the current simulations) and the inner pressure of the cell is removed so that the cell collapses and its area gradually reduces until a prescribed minimal value is reached. At this time, the cell is considered to be dead and will be removed from the system. These stages of cell apoptotic death are represented in our model by a gradual reduction in cell area and changes in shape as shown in Figure 7.

#### Cell polarization

The membrane points are devised in such a way that they can be specified independent of each other. Hence, the properties of each point and its corresponding subunits can be controlled. That is, the points can be categorized into two or more subgroups. By changing their properties independently, apical, basal and lateral regions in our model can be easily defined.

### c. ECM

**Figure 8:**
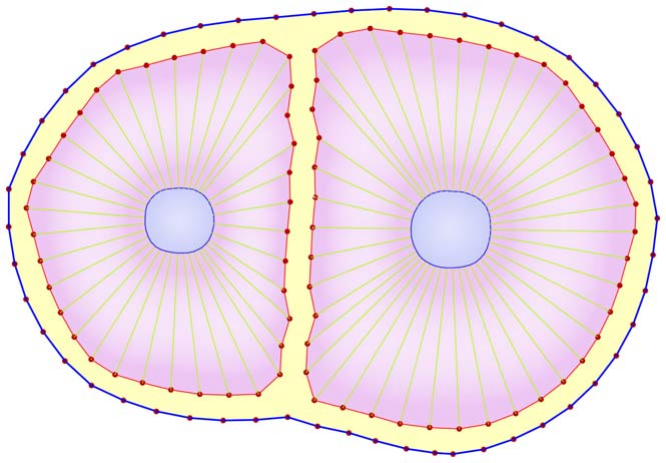
ECM. In 3D culture, our model allows for the investigation of a cross section of the system and the cells that are immersed in ECM. In 2D, the contact region of the ECM and the cell is a line that surrounds the cell; therefore, an enclosed curve (or ring) can be used for the ECM that surrounds the cells (blue curve). The ECM is modeled using a chain of subunits connected in series, where each point connects two subunits (blue curve with red points). These subunits can interact with cell points in a manner similar to the interaction of two points of different cells. This chain is flexible and the number of its corresponding points can be increased or decreased.

Before the interaction of the cell with its environment can be investigated, the extracellular matrix (ECM), which is a complex structural entity surrounding and supporting cells that are found within mammalian tissue must be modeled. The ECM is often referred to as connective tissue and the cells can connect to the ECM via adhesion receptors. Two methods can be defined for modeling the ECM; in a 2D culture, the ECM or substrate is the area under the cells, hence the cells interact with the ECM by adhesions and adhesions are controlled by a “drag coefficient”, so the drag coefficient 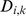 can be related to 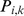. As mentioned before, referring the drag coefficient to the adhesion intensity is used in previous models such as [62]–[64], so in the following, if a 2D culture is modeled, the adhesion intensity is equivalent to the drag coefficient. In 3D cultures, our model allows for investigations of a cross section of the real system and the cells immersed in the ECM. In 2D, the contact region of the ECM and cell is a line that surrounds the cell, so an enclosed curve (or ring) can be used for the ECM that surrounds the cells. The ECM is modeled using a chain of subunits connected in series (Figure 8), where each point connects two subunits. These subunits can interact with cell points in the same manner as the interaction of two points of different cells. Depending on the model, the cells are embedded in the outer region of the ECM ring or the inner region. This curve is flexible and the number of its corresponding points can be increased or decreased. The drag coefficient property of each membrane point indicates the interaction of the cell with other cells or with the ECM, which is situated perpendicularly to the cross-sectional region. The model can also incorporate a cellular automata model for the ECM, allowing for the investigation of the diffusion of mobile receptors and signals in the ECM.

### d. Environmental effect

#### Cell-cell and cell-substrate interaction

**Figure 9:**
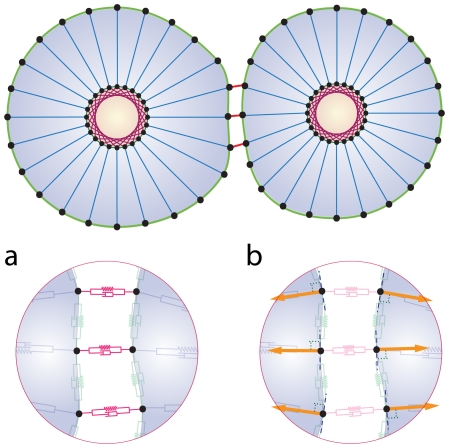
Cell-cell interaction. Each cell can interact with another cell and substrate in two methods, adhesion and/or repulsive forces due to elasticity, as shown in Eq. (11). a) In this model, all points located on the cell membrane serve as potential sites of cell-cell connections. Each two points from different cells or cell-substrates are connected via a Voigt subunit, once they are closer than a determined value of 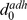. The adhesion subunit parameters between two cells are a function of these parameters, for more detail see Eq. (12). b) The repulsive force acts as a short range force. It is a passive force resulting from the elastic interaction with neighboring cells and acts on each point of the cell, when the distance to the other cell points or substrate is less than 
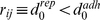
. The magnitude of the repulsive force is a function of the distance of two surfaces (Eq. (13)) and its direction is perpendicular to the membrane, pointing inward to the inner cell.

Each cell can interact with other cells and substrates in two methods, adhesion and repulsive forces due to elasticity (Figure 9).
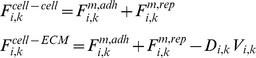
(11)Cells are not often found in isolation, but rather tend to stick to other cells or non-cellular components of their environment. They usually bind directly to one another through cell-surface proteins that form specialized cell-cell junctions. These cell adhesive properties are especially important in epithelial tissues since they constitute barriers between different body compartments.

In our model, all points located on the cell membrane serve as potential sites of cell-cell connections, which can be transformed to either adherent or repulsive forces. Here, simple rules can be considered for the formation of cell adherents and tight junctions that depend only on the cell phenotype and on the distance between neighboring cells, that is whether or not the membrane receptors of one cell fall into the minimum distance, 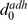, of another cell. Gap junctions and chemical signals are not explicitly included, but it can be assumed that cells are able to communicate and signal information with neighboring cells. Each of the two points from different cells or cell-substrates connect each other with a subunit, if they are within a minimum separation distance of 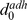 (Figure 9a).

For each cell, specific parameters for subunit attachment are assigned, making the adhesion subunit parameters between two cells a function of these parameters. For adhesion between 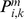 and 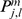,it can be said:
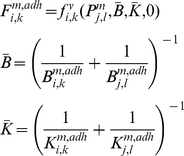
(12)Where 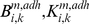 are the attachment subunit parameters of 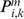 and 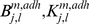 are the attachment subunit parameters of 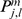. When the cells are pulled apart, they deform, but points remain stuck until a distance 
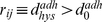
 at which point their contact ruptures, displaying typical hysteresis behavior. It must be mentioned that sometimes, for example, in apoptosis, the adhesion can be disrupted unilaterally by one cell without satisfaction of the above condition.

A repulsive force acts as a short range force. It is a passive force resulting from the elastic interaction with neighboring cells and acts on each point of the cell where the distance to the other cell points or substrate falls shorter than 
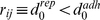
. The magnitude of the repulsive force is a function of the distance of two surfaces and its direction is perpendicular to the membrane, pointing inward to the inner cell (Figure 9b).
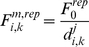
(13)Where 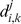 is the nearest distance between 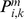 and the membrane surface of 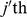 cell or ECM.

#### External field

The cell can be subjected to an external force, such as pulling forces by the surrounding tissue or under certain fields, e.g. electromagnetic fields. This can be represented by adding an external force 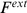 to each point on the cell membrane.

### e. Justification for the use of Voigt elements

It should be noted that although the individual Voigt elements are simple, they combine to form a complex system (Figures 1,2,3 and 8) with dynamics that are beyond the simplicity of individual linear elements. Although our model is composed of a network of linear Voigt elements, the behavior and dynamics of these elements are not linear for several reasons. First, model parameters such as spring constants and viscosity coefficients change dynamically through time based on mechanical and biochemical signaling as well as the state of the cell as previously mentioned. Given that these parameters change dynamically, the elements can no longer be considered linear. Second, during the cell cycle some constant parameters are changed dynamically. For example, in cell growth when a point is added to the membrane, the rest length of radial springs is increased in proportion to 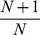. During apoptosis the parameters change dramatically as previously mentioned, the rest length of each subunit spring is reduced resulting in the cell collapsing and its area gradually decreasing and during motility, the cytoskeletal subunit parameters of these elements will change periodically in a coordinated fashion to generate movement. To further establish the non-linearity and finite-extensibility of elements, the overall number of elements and points changes accordingly during growth and mitosis while during adhesion relevant elements can be created or destroyed based on the distance between two points (on different cells/ECM) which shows that elements are neither linear nor infinitely extensible in all cases. Finally, in the process of developing this model, we felt that the input parameters should best represent those determined through experimental methods. As a result, we selected those established by Coskuna et al. [34] for Voigt elements rather than using arbitrary or speculative parameters for more complex element types. However, the flexibility of this model allows for easy implementation of more complex building blocks such as Maxwell elements, given valid, experimentally derived element parameters.

We acknowledge that the Voigt element is simpler than other element types, however, as our results show; a model based on Voigt elements is capable of reproducing cell behavior. We believe as a rule that as long as a simple model satisfies our interest and can reproduce desired behavior, there is no advantage to increasing model complexity.

## Results and Discussion

### a. Case studies from relevant biological examples

#### Monolayer cell culture and effect of adhesion intensity

It is well documented that cell shape, proliferation, and ECM are important aspects of cell culture [70]. The adhesion between cells and ECM can dramatically affect invasion of tumor cells and the quality of the epithelial monolayer of the cell. The first application of our model investigates this phenomenon by simulating a monolayer culture of cells and the exploring the effects of adhesions on tissue formation and morphology. The process began from two cells, which were allowed to reproduce freely while subject to ideal conditions that were suitable for proliferation, without the occurrence of apoptosis.

**Figure 10:**
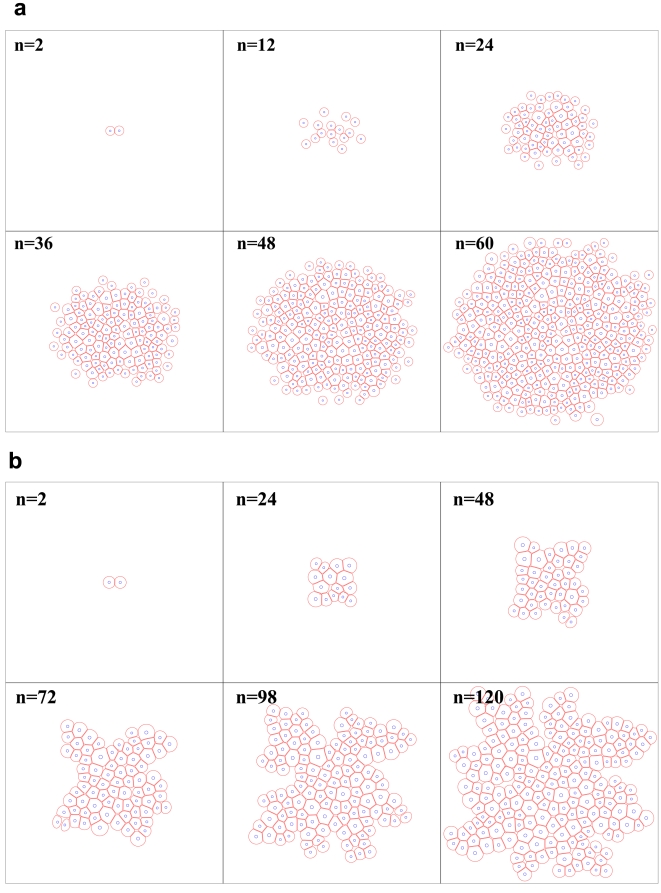
The monolayer culture of cells and the effects of adhesions on tissue formation and morphology. a) In this simulation, D = 0.0001*D0 i.e. low intensity of adhesions. We began from two cells and allowed them to reproduce freely, subject to conditions that are suitable for proliferation without the occurrence of apoptosis. Results show a filled circular culture and fast proliferation. n represents the dimensionless elapsed time. b) D = D0 i.e. high intensity of adhesions. The process began from two cells which were allowed to reproduce freely. The results show dendrite morphology for the culture which can be seen frequently in tumors. It is conceivable that this special morphology is due to strong adhesion between cancer cells and the ECM. The process has a slower proliferation rate than part a. The epithelial cells in a monolayer appear as polygonal cells. It also can be seen that the average number of neighbors for any cell is 6, regardless of the value of the drag coefficient.

Typical snapshots of proliferation under two different adhesion intensities are shown in Figure 10. As the figures show, adhesion intensity plays an important role in the morphology of each culture. At low adhesion intensities (low drag coefficients) a filled circular culture can be seen, whereas high adhesion intensities (high drag coefficients) tend to show dendrite morphology, which can be seen frequently in tumors. It is conceivable that this special morphology is due to strong adhesions between cancer cells and the ECM. Our results suggest that increased adherence may lead to decreased culture growth; however, this result is not always supported by experiments. This anomaly may be explained by the fact that our model only incorporates the biomechanical behavior of the cell, whereas there is some signaling pathway that stimulates the growth factors when cells adhere to substrate. Possible methods for incorporating biochemical effects into the model are discussed later in this paper.

**Figure 11:**
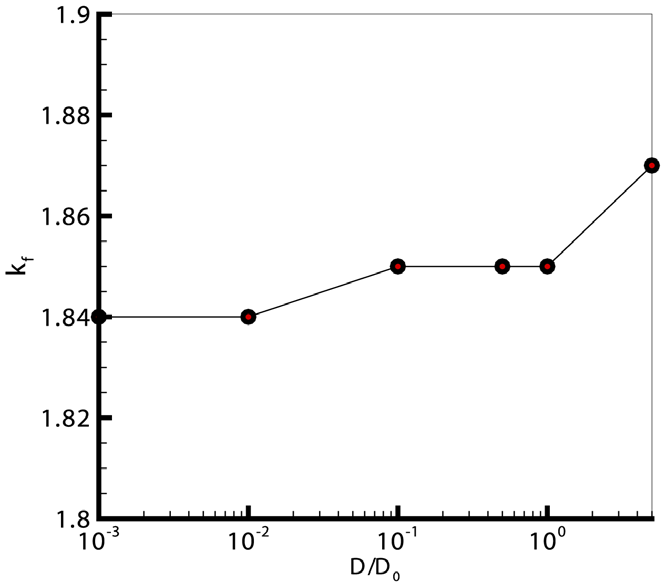
Effect of adhesion intensity on monolayer cell culture properties. The value of 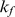 for cultures with 120 cells, compared against the adhesion intensity. This graph shows a linear relation between 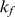 and the adhesion intensity, which in turn suggests that the cellular shapes are almost circular at low drag coefficients, and diverge from being circular as the drag increases.

To characterize the geometry of the cells in a culture, a form factor, 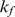, was introduced [71]:
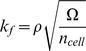
(14)where 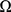 is total occupied area, 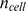 is number of cells, and ρ is cell edge density, which is given by:
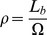
(15)in which 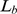 is the sum of the lengths of all internal cell boundaries plus half of the perimeter of the patch; it is assumed that these edges are to be shared between the patch shown and a mirror image patch that adjoins them. Physically, ρ corresponds to the area density of the cell edges. Minimum value of 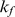, 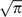, occurs when the cell has a circular shape. The value of 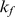 for cultures with 120 cells against adhesion intensity is shown in Figure 11. This shows a linear relation between 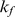 and the adhesion intensity, which in turn suggests that the cellular shapes are almost circular at low drag coefficients, and diverge from circular shape as the drag increases.

On the other hand, the epithelial cells in a monolayer appeared as polygonal cells. It also can be seen that the average number of neighbors for any cell is 6, regardless of the value of the drag coefficient.

#### Growth of epithelial cells and interaction with the ECM

**Figure 12:**
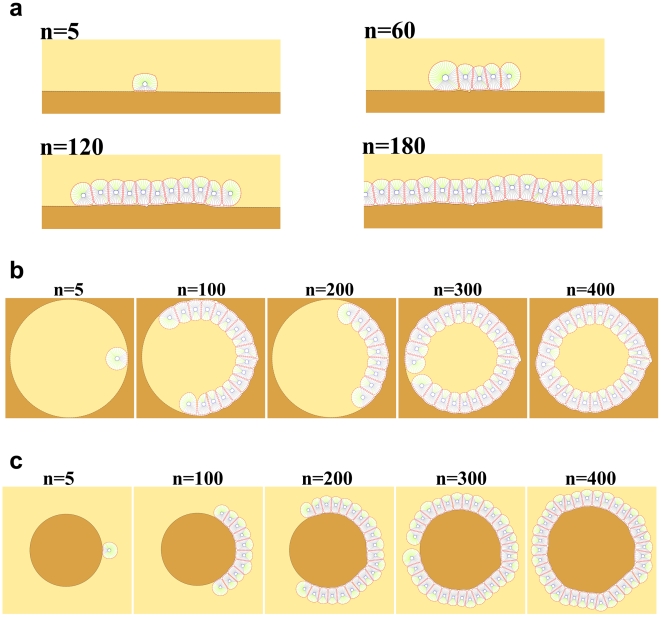
Growth of epithelial cell interacting with the ECM. In this simulation a cross-sectional perspective of cell culture can be seen. Therefore, the ECM is a line of points to which the cells adhere. These points are dynamic and can change if needed. n represents the dimensionless elapsed time. a) Cells plated on a layer of surface culture. As the cell proliferates, a stable, uniform monolayer will be constructed. The axis of division is perpendicular to the ECM, likely related to polarized cells. If a cell detaches from the ECM due to the loss of polarity, it will activate the apoptosis pathway. b) A hole exists in the ECM, where a cell is located for attachment and proliferation. After polarization, the cell starts to proliferate and create a stable, lumen-containing cyst, lined by a single layer of epithelial cells. As it can be seen, the ECM is deformed a bit due to the dynamic interaction between the ECM and cells during the growth process. c) Shows an inverted cyst. A circular ECM is located in a suspended culture, to which a cell is attached and polarized. Upon completion of proliferation, cells surround the entire surface of the ECM and create inverted cysts, with matrix deposited on the inside of the cyst. If the process is allowed to continue, the cyst will grow further and become greater, which corresponds to a bigger ECM. This is because the volume of ECM in our model can freely increase.

In this simulation, a cross-sectional perspective on cell cultures can be seen where the ECM is a line of points to which cells adhere. These point numbers are dynamic and can change if needed. Most living tissues are typically separated from the exterior by a delimiting interface of epithelial cells. These layers of cells align with the cavities and surfaces of structures throughout the body. Epithelial cells can take any shape and can be classified by their shape or by the function of the cell where they are located. They can take shape as squamous layers or monolayers and these layers can be folded into circular acini or ducts. For example, epithelial cells that are found in the thyroid or cornea of the eye function in aligning fluid-filled lumens [72]. Mature epithelial cells are highly polarized with separate apical and baso-lateral membrane compartments. The basal cell surface is attached to ECM material and in most epithelia the opposite apical surface is free from an apposed extracellular layer. From the histological organization of epithelium, it shows that attachment to the ECM is essential in polarization, and plays an important role in directing the polarity. Observation of epithelial cell growth and morphogenesis in different environments show that morphology *in-vitro* depends on both the structure and composition of the external environment of the cells. The main morphological distinction in 3-D embedded cultures is the formation of cysts, i.e. the stable, self-enclosed monolayers. In suspension cultures, epithelial cells form cysts as well, but the epithelial cells adopt an inverted polarity, laying down basement membranes on the inside of the cyst [1], [3]–[4]. In plane culture, epithelial cells normally constitute a smooth monolayer covering the whole ECM surface like a wrap. Each of these three categories is a representative of the growth of different epithelial cell types *in-vitro*, which have a common morphological scheme [72]–[73]. This suggests that the presence and relative locations of cells, ECM, and matrix-free (or cell-free) space are very essential for the expected behavior of epithelial cells. Cells plated on a layer of surface culture construct a stable, uniform monolayer as they proliferate (Figure 12a). The axis of division is perpendicular to the ECM, likely related to polarized cells. If a cell detached from the ECM due to the loss of polarity, it will activate the apoptosis pathway. In a different case (see Figure 12b) a hole exists in the ECM, where a cell is located and allowed to attach and proliferate. After polarization it starts to proliferate and creates a stable, lumen-containing cyst, lined by a single layer of epithelial cells. As it can be seen, the ECM is deformed a bit due to the dynamical interaction between the ECM and cells during the growth process. Figure 12c, on the other hand, shows an inverted cyst. A circular ECM is located in a suspended culture, to which a cell attaches and is polarized. Upon completion of proliferation, cells surround the entire surface of the ECM and create inverted cysts, with matrix deposited on the inside of the cyst. If the process is allowed to continue, the cyst will grow further and become larger, corresponding to a bigger ECM. This is due to the fact that the volume of the ECM in our model can freely increase.

#### Effects of gap in ECM

**Figure 13:**
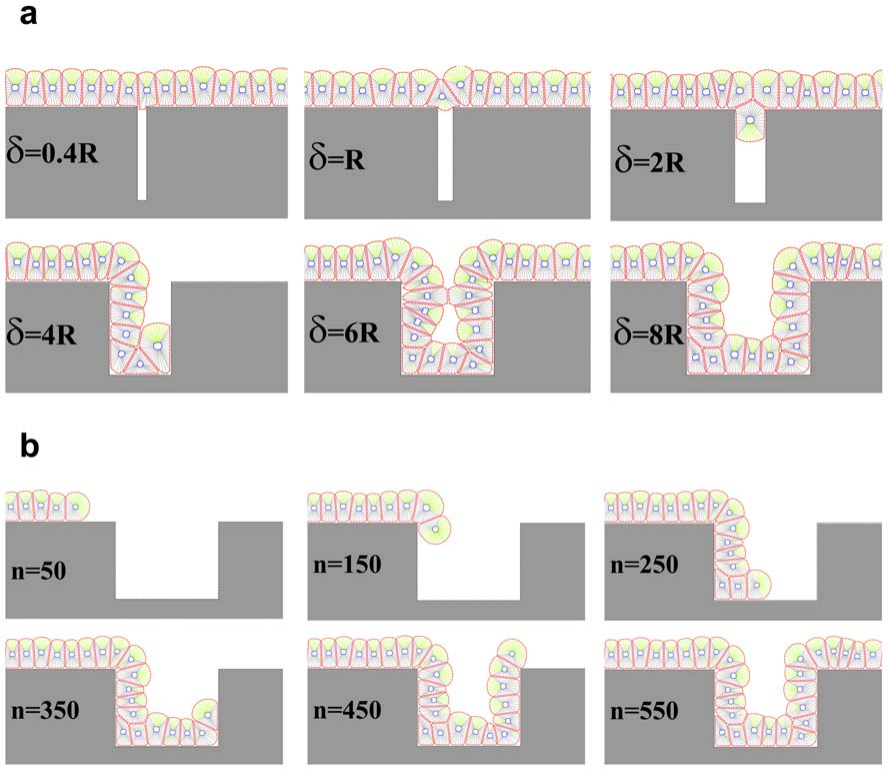
Effects of a gap in ECM surface. ECM is considered to be rigid and not affected by cells; however, cells still adhere to ECM and are polarized. a) Figure shows the final results for various gap sizes. If the gap width is denoted with 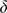 and the radius of a free epithelial cell with 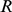, then it can be seen that cells cannot line the gap for 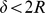. For 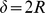 the first cell which meets the gap will enter it, although due to the pressure of the walls it would not be able to continue its growth and division, so it fills the entry and blocks the gap. Other cells pass over the gap and again create a linear monolayer. For 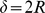 cells cannot ignore the gap and penetrate it. They continue their proliferation into the gap; however, when they reach the internal right corner, because of the limitation in space and the forerunning cells being subject to direction changing, the growth is stopped and the cells are entrapped in the gap. For 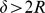 the cells can enter the gap without any problem and line it. b) A few snapshots of the growth process when the gap is equal to 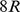. n represents the dimensionless elapsed time. During simulation, cells show differing behaviors at the corners. The growth rate of cells decreases at the internal corner and increases at the external corner. In addition, at external corners, due to the sudden decrease in contact area, cells detach from the ECM more easily in response to the pressure of neighboring cells. This, in turn, leads to loss of polarity and apoptosis.

As a different test case, the effects of gaps in the ECM on the formation of a confluent epithelial monolayer are investigated. The ECM is considered to be rigid and not affected by cells; however, cells still adhere to the ECM and are polarized. Figure 13a shows the final results for various gap sizes. If the gap width is denoted as 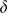 and the radius of a free epithelial cell as 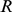, then it can be seen that cells cannot line the gap for 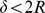. For 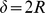 the first cell who comes into contact with a gap will enter it, although due to the pressure of the walls, it would not be able to continue its growth and division, so it fills the entry and blocks the gap. Other cells, accordingly, pass over the gap and again, create a line monolayer. For 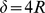 cells could not ignore the gap and penetrate it. They continue their proliferation interiorly, however, when they reach the internal right corner, as the forerunning cells are subject to direction change due to the limitation in space, the growth is stopped and the cells are entrapped in the gap. For 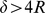 the cells can enter and line the gap without any problem. It must be mentioned that in all of the above simulations, cells show different behaviors at the corners. The growth rate of cells decreased at the internal corner and increased at the external corner. In addition, at external corners, due to the sudden decrease in contact area, cells detach from the ECM more easily in response to the pressure of neighboring cells. This, in turn, causes the loss of polarity and apoptosis. Figure 13b shows snapshots of the growth process when the gap is equal to 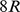.

#### Tensegrity and Tissue Morphogenesis

Studies on the mechanisms of epithelial morphogenesis and tubulogenesis have revealed that local changes in ECM structure and its mechanics play essential roles in tissue structure and remodeling. Using the tensegrity-based architecture, a mechanical model of cell structure explains how local changes in ECM mechanics may guide tissue patterning according to that model [47], [74]. It has been speculated that up-regulation of the ECM due to local thinning within the ECM can lower any stiffness that may occur. This in turn causes the surrounding cells to apply tractional forces, thereby, causing an increase of forces between the cell-ECM receptors causing changes within cell shape and morphology. Therefore, based on this vision, epithelial cell growth and relocation are restricted to particular groups of cells that adjoin the thinned region. Outward budding occurs when these cells extend and grow. Other cells along the same ECM do not experience this stress and, therefore, remain inactive.

**Figure 14:**
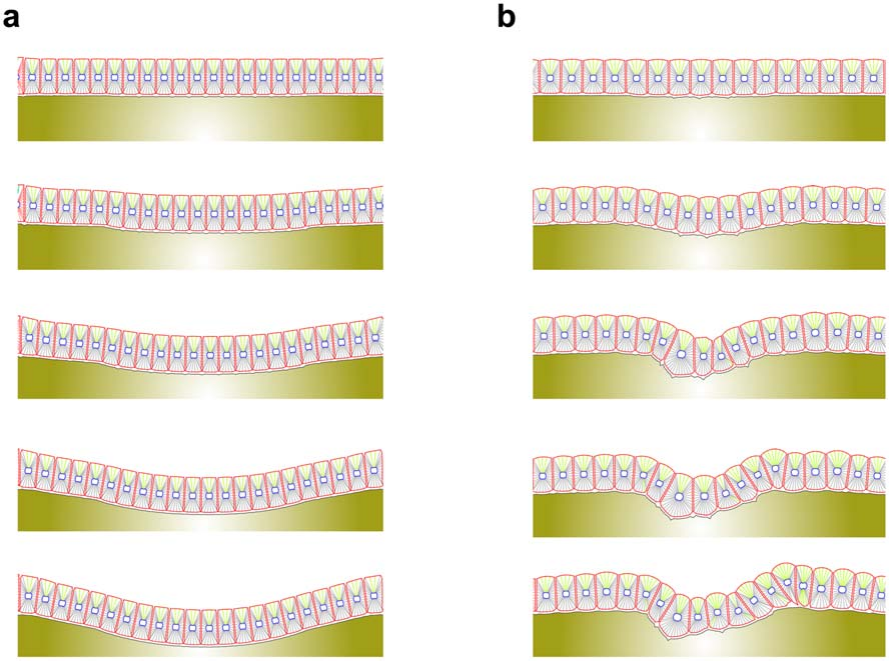
Tensegrity and Tissue morphogenesis. At this stage, an attempt is made to model the tensegrity hypothesis. To do so, a monolayer of epithelial cells is placed on the ECM. This causes the ECM to get thinner, by decreasing the drag coefficient at the center of the ECM (color gradient represents change in drag coefficient). The simulation is run with two densities of cells. The cells at this region have localized growth and motility, which drive the ECM downward. As a result, cells find space for more growth and proliferation. Therefore, they continue to drive the ECM further, which finally leads to the creation of a bud. This bud can be the first stage of tubulogenesis. a) Due to the high density of cells, they do not have enough space to grow and proliferate, so there is more organization and less deformation. b) Shows proliferation and less symmetry due to low cell density.

At this stage, an attempt is made to try to model this hypothesis. To do so, a monolayer of epithelial cell is placed on the ECM and thinning of the ECM occurs by decreasing the drag coefficient at the center of the ECM (showed in Figure 14 in white). The program runs with two densities of cells, as shown in Figure 14. The cells at this region have localized growth and motility, which drives the ECM downward. As a result, cells find space for more growth and proliferation. Therefore, they continue to drive the ECM further, which finally leads to the creation of a bud. This bud can be the first stage of tubulogenesis. In Figure 14 due to the high density of cells, there is not enough space for the ECM to grow and proliferate, so there is more order and less deformation. But as can be seen in Figure 14b there is proliferation and less symmetry, due to low cell density.

#### Formation of hollow epithelial acini

**Figure 15:**
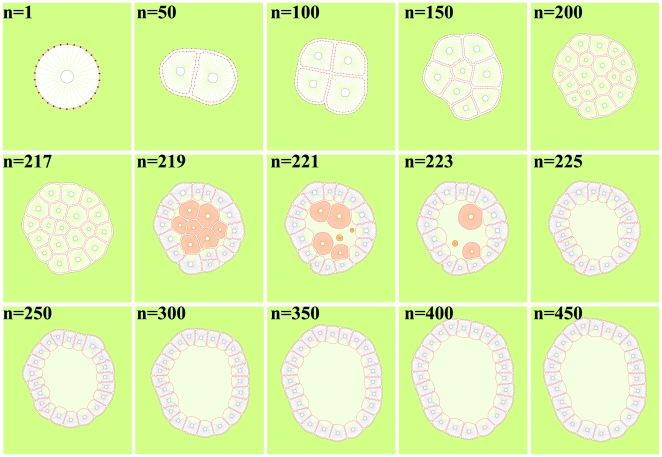
Formation of hollow epithelial acini. This stage shows modeling snapshots of self-arrangement of individual eukaryotic cells into a stable hollow acinar structure. In this model a single cell (n = 1) undergoes several consecutive divisions and gives rise to a small cluster of cells containing two different populations (n = 100–217): the inner cells are entirely surrounded by other cells which do not have access to ECM, and the outer cells partially face the ECM. Further cell proliferation leads to the expansion of the whole cluster. During this stage (n = 150–217) some intercalation of outer cells to the interior for the preservation of the circular shape of the tumor can be seen. As the intensity of adhesion between cells increases, the process of intercalation becomes more difficult. After this stage, when the tumor reaches a certain age, the cell undergoes further differentiation of outer cells and results in their apical-basal orientation and self-arrangement into one layer of polarized epithelial cells of regular cubical shapes (n = 219) and the inner cells are then triggered by polarized cells to enter the process of cell apoptosis. Each cell that does not have access to the ECM and as a result does not get polarized will die (n = 219–225). This process leads to the creation of the inner lumen. Consequently, the proliferation of polarized cells is suppressed and the final structure stabilizes in the form of a hollow epithelial acinus(n = 250–450). Moreover, the processes of cell proliferation, polarization and apoptosis need to be well coordinated in order to maintain the hollow acinar structure in a stable manner; otherwise cell overgrowth may lead to intraductal carcinomas. This coordination shows that this process is very dependent on biochemical signaling between cells. The final shape of the tumor is very dependent on the viscosity of the ECM. If the viscosity of the ECM is high enough (i.e. drag coefficient is high in our model, due to this process trying to move during a minimal distance in energy space), the cell maintains its circular morphology. However, if the viscosity of the ECM is reduced, the cell deviates from the circular shape.

The epithelial acini are experimental culture structures that help to explore the detailed mechanism underlying epithelial cancers *in-vitro*, [2], [75]. As discussed earlier, well developed epithelial acini are composed of one layer of closely packed epithelial cells covering the hollow lumen. Examples of such cell culture systems that cultivate in vitro in a form of cysts or acini are Madin–Darby canine kidney (MDCK) cells and mammary epithelial MCF-10A cells [1]–[2]. The details of formation of acinar structures are not completely understood. In general, the mechanism of obtaining an acinar structure is similar in all types of cells. This process begins with a single cell planting itself on the suitable media culture. This pioneer cell starts growth and proliferation to form a small 3-dimensional collection of randomly oriented cells. These cells can be divided in two distinct groups. The first group consists of a surface layer of cells in direct contact with the ECM and the second group is internal cells enclosed entirely by other cells. They do not have any direct contact with the ECM. To continue acini development, cells in the outer layer are polarized and inhibit an asymmetry in an apical-basal surface and become insensitive to proliferative signals. Differentiation of outer cells coincides with the start of the apoptosis pathway of inner cells. As a result, the hollow lumen is formed and the acinar structure remains hollow [2], [75]. In this stage, an attempt is made to model self-arrangement of individual eukaryotic cells into a stable hollow acinar structure. In this model, a single cell (Figure 15, n = 1) undergoes several consecutive divisions and gives rise to a small cluster of cells containing two different populations (Figure 15, n = 100–217): the inner cells entirely surrounded by other cells which do not have access to the ECM, and the *outer* cells partially facing the ECM. Further cell proliferation leads to the expansion of the whole cluster. During this stage (Figure 15, n = 150–217) intercalation of outer cells to inner cells (or inward) for preservation of the circular shape of the tumor can be seen. It should be noted that if the adhesion between cells is stronger the process of intercalation is less prevalent. After this stage, when the tumor reaches a certain age, the tumor undregoes further differentiation of outer cells which results in their apical-basal orientation and self-arrangment into one layer of polarized epithelial cells of regular cubical shapes (Figure 15, n = 219). The inner cells are then triggered by polarized cells to enter the apoptosis pathway. As a result, each cell that does not have access to ECM, and is therefore not polarized, will die (Figure 15, n = 219–225). This process then leads to the creation of an inner lumen. Consequently, the proliferation of polarized cells is suppressed and the final structure stabilizes in the form of a hollow epithelial acinus (Figure 15, n = 250–450). Moreover, the processes of cell proliferation, polarization and apoptosis need to coordinate well in order to maintain the hollow acinar structure in a stable manner. Otherwise overgrowth of the cell may lead to intraductal carcinomas. This coordination shows that this process is very dependent on proper biochemical signaling between cells. The final shape of the tumor is very dependent on the viscosity of the ECM. If the viscosity of the ECM is high enough the tumor attempts to keep a circular morphology. If the viscosity of the ECM is reduced, the tumor deviates from the circular shape.

### b. Conclusion

A biomechanical, cell based model was developed that describes both individual cell behavior and cell-environment interaction based on cellular mechanics. The model has the ability to simulate the global and local mechanical characteristics of the single cell. Each cell in this model is an individual unit containing several subcellular elements, such as the plasma membrane, enclosed by viscoelastic elements that play the role of cytoskeleton, and the viscoelastic elements of the cell nucleus. The cell membrane is divided into segments where each segment incorporates the cell's interaction and communication with its environment, such as adherens junctions. These deformable cell models can mimic many aspects of real cells such as growth, cell division, apoptosis and attachment to other cells or ECM. It was shown that these cell models can mimic various topologies of tissue such as cyst or tumor or monolayer. In addition, it was demonstrated that the model is capable to describe such phenomena such as interaction of a culture with a geometrical gap in substrate or buds. This model offers utility to investigate the role of individual cells as a part of tissue and how the property of each individual cell may affect the mechanical and morphological property of the tissue. The model makes it possible to investigate mechanical and physical behavior of different tissue in cell scale in various mechanical conditions. The structure of the model is simple and is based on a small number of parameters, allowing for high performance computing of large cell populations in a reasonable time. One of the important aspects of the model is ability to simultaneously investigate the intra- and extra-cellular biomechanical behavior. By changing the model parameters, it is possible to apply the model to different types of cells and investigate their interaction in different cellular constructs. This model is in the first stage of its life and needs many improvements, for example finding the quantitative parameters for different cell types or improving the inner cell structure such as the nucleus. Most experimental investigations employ a two-dimensional substrate; however, to gain further insight into the behavior of epithelial cells *in-vivo*, we must switch from 2D to a 3D model. With this modification, we can investigate the biomechanical effects in a 3D environment. For this purpose, we must define a 3D cell and rather than a 1D curve for the cell membrane we will have a surface and a network of nodes. Our model can be extended to three dimensional space in a straightforward manner. All equations can be carried over to 3D space without significant changes. A more complex network of nodes and elements may be needed to define the cell structure, but all additional boundary points and forces can be incorporated into the 3D model analogous to methods presented in this paper. The algorithms defining cell processes can also be carried into 3D space with some changes.

One of the biggest challenges in moving from 2D to a 3D model is an increase in the number of total nodes which results in a dramatic increase in the computational cost. For a cell membrane with radius *r* and *N* discrete points of length *dr* we have:
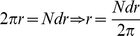
If we move to a 3D environment where each surface *dr^2^* contains a single point from the set of points *N'*, the number of points on the surface of the membrane will increase to:
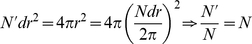
The ratio between the number of points in 3D compared to the number of points in 2D will be 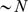. Since an additional element (z) is added in 3D, the computational cost will be increased by 50%. Additionally, each point will be connected to 4 other points of the surface rather than only 2 points in 2D and as a result, the computational cost will be increased by a total of *6N* times. In our case studies, where the initial number of points of each cell was 40, we would expect computational time to increase 240 times that of the original. It should be mentioned that the majority of challenges in transitioning from a 2D model to a 3D model are related to computational obstacles and associated programming techniques rather than biological or physical concepts.

Another major step in developing the model in the future is importing the biochemical aspect of the cell into this model. Cells can respond to a variety of environmental cues in a dynamic environment. These cues can be biochemical or mechanical in nature and can lead to changes in cell function and phenotype, both under normal physiological conditions and in pathological states. Most of the cells are surrounded by a highly complex ECM that is important in maintaining tissue structure but also plays key roles in guiding cell function. Cells bind to the ECM via specific integrin receptors and this binding can directly affect cell function. Furthermore, other signals that a cell receives from its environment are transmitted through and modulated by the ECM. Biochemical signals (e.g., ions, small proteins, or growth factors) must pass through the ECM and in some cases are sequestered and released by the matrix. Mechanical signals (e.g., tensile, compressive forces, or shear forces) are also transmitted by the ECM to the cell via integrin receptors that link the external environment to the cytoplasm and cytoskeleton.

Cell function is regulated by the entirety of the cellular environment, including cell-cell interactions, ECM components, humoral factors, local chemical conditions, and mechanical forces. In vivo and in vitro studies have the advantage that they maintain this complex environment, but the large number of variables that are difficult to control makes it challenging to isolate specific effects in experimental studies. In silico studies have the advantage that treatment variables can be controlled.

The nodes on the membrane can play the role of receptors allowing us to numerically insert chemotactic signals in our model and to use the reaction diffusion system for external signals. With knowledge about the internal biochemical pathway, we can model the biochemical properties of our model. One foreseen challenge in this work is that physical forces play a critical role in cell integrity and development, but little is known regarding how cells convert mechanical signals into biochemical responses [76]. Some molecules like integrins, focal adhesion proteins, and the cytoskeleton in the context of a complex cell structure—when activated by cell binding to the ECM—associate with the skeletal scaffold via the focal adhesion complex. Vinculin is presented as a mechanical coupling protein that contributes to the integrity of the cytoskeleton and cell shape control, and examples are given in literature of how mechanical signals converge into biochemical responses through force-dependent changes in cell geometry and molecular mechanics [77]–[81].

In addition to the discretized approach for modeling the cell object we should use another distinct approach for discretizing the chemotactic signals. For signaling we will use cellular automata (cellular automata can be viewed as spatially extended decentralized system made up of number of individual components and may serve as simple model of complex systems. According to this interpretation, the CA can be traced back to biological modeling especially in reaction diffusion systems). The interaction of two distinct discretized models, i.e. cell and chemotactic signals are very important and require additional effort.
